# Paronychia an Epidemic

**Published:** 1852-03

**Authors:** James E. Morgan

**Affiliations:** Demonstrator of Anatomy in National Medical College, Washington, D. C.


*Paronychia an Epidemic.* By James E. Morgan, M. D., Demonstrator of Anatomy in National Medical College, Washington, D. C.—Paronychia has, without doubt, existed in Washington this summer as an epidemic; and, as its prevalence in that form has not, to my knowledge, been noticed by any writer, I have thought that an account of it might not be uninteresting to the profession. Scarcely a day passes but that I am called upon to prescribe for several cases of this apparently trifling, but always painful, and sometimes fatal disease.

It usually commences with a pain in the arm and forearm, which continues for two or three days with greater or less intensity, according to the severity of the case. These symptoms then suddenly cease, and the patient is seized with a soreness like the pricking of a needle, or brier, in one or all the fingers. This continues to increase in severity; the fingers and hand become very much swollen, the pain is almost insupportable, and the patient is worn out with suffering, without being able to sleep or rest. Constitutional symptoms at last supervene. I

have seen fever, loss of appetite, debility, and spasmodic asthma, terminating in death, follow successively in a few days.

*Case.*—James Sumby, colored, a brickmaker, ætat 32, large frame, in general enjoyment of good health, was taken on the 3d of July, with a pain in the arm, followed by paronychia of the worst form. I was called to see him on the 8th, and found him with some fever, tongue dry and slightly coated, pulse nearly natural, his finger and hand much swollen, and suffering great pain. I made a free incision into his finger, from which a large quantity of blood and pus was discharged. Ordered a flaxseed meal poultice to be applied to the hand, and ten grains of Dover's powder to be taken at night. On the 9th, I was sent for in great haste, and fouud him sitting erect in a chair, with great difficulty in breathing, mucous râle, and inability to lie down or expectorate. The countenance was hippocratic and anxious; pulse quick and scarcely perceptible; extremities cold, and the whole surface suffused with a clammy perspiration. These symptoms, in a milder form, had made their appearance in the preceding night. I ordered the following: ℞. Tr. valerian vol. ℥ss; lac. ammoniac ℥iss; lac. assafœt. ℥ij. M. A tablespoonful every two hours in a wineglass-full of strong brandy toddy. He continued to grow worse, and died that night.

*Autopsy* twelve hours after death. Abdominal viscera healthy, and of normal size; heart natural; the bronchiæ and air-cells of the lungs, which crepitated to the touch, were completely filled with a bloody, tenacious mucus. I regarded this as a case of spasmodic asthma, caused by the same pathological condition of the pneumogastric nerve which exists in the spinal nervous system in tetanus, its mediate cause being paronychia.

It seems strange, at first thought, that paronychia should become epidemic; but when we reflect that so little is known of the laws that govern the existence and propagation of epidemics, and that many diseases belonging to this class, such as furuncles, carbuncles, erysipelas, &c., are sometimes epidemic, it ceases to create surprise.

I regard paronychia as a furuncle, modified in its symptoms by the structure of the seat of its attack. The violent pain is imputed by writers to the hard and unyielding nature of the skin on the finger; but I presume that most practitioners have seen the skin, in this disease, stretched as much as it was possible for the parts under it to require even in the most intense inflammation. Some other cause, therefore, must produce the pain. I attribute it to the large number of nervous fibres distributed to the ends of the fingers.

Professor Weber has shown, in his experiments on the the sensibility of different parts of the skin, that the nervous fibres on the points of the fingers are one-third of a line apart; while those on the back, neck, and nates (the usual seat of boils), vary from ten to thirty lines. Supposing pain to be in proportion to the number of sensory nervous fibres, the greater suffering in paronychia is readily understood. In other respects, whitlows differ from boils; and the fact of both prevailing at the same time, and even in the same person, seems to go far in proving their identity.—*Ibid.*

